# Decreased Human Platelet Activation and Mouse Pulmonary Thrombosis by Rutaecarpine and Comparison of the Relative Effectiveness with BAY11-7082: Crucial Signals of p38-NF-κB

**DOI:** 10.3390/molecules27020476

**Published:** 2022-01-12

**Authors:** Wei-Chieh Huang, Shaw-Min Hou, Ming-Ping Wu, Chih-Wei Hsia, Thanasekaran Jayakumar, Chih-Hsuan Hsia, Periyakali Saravana Bhavan, Chi-Li Chung, Joen-Rong Sheu

**Affiliations:** 1 Graduate Institute of Medical Sciences, College of Medicine, Taipei Medical University, Taipei 110, Taiwan; d119110003@tmu.edu.tw (W.-C.H.); cgh05414@cgh.org.tw (S.-M.H.); mpwu@mail.chimei.org.tw (M.-P.W.); d119106003@tmu.edu.tw (C.-W.H.); jayakumar@tmu.edu.tw (T.J.); 2 Department of Cardiovascular Center, Cathay General Hospital, Taipei 106, Taiwan; 3 School of Medicine, College of Medicine, Fu Jen Catholic University, New Taipei City 242, Taiwan; 4 Division of Urogynecology, Department of Obstetrics and Gynecology, Chi Mei Medical Center, Tainan 710, Taiwan; 5 Translational Medicine Center, Shin Kong Wu Ho-Su Memorial Hospital, Taipei 111, Taiwan; T014913@ms.skh.org.tw; 6 Department of Zoology, Bharathiar University, Coimbatore 641046, Tamil Nadu, India; bhavan@buc.edu.in; 7 Department of Internal Medicine, Division of Pulmonary Medicine, Taipei Medical University Hospital, Taipei 110, Taiwan; 8 School of Respiratory Therapy, College of Medicine, Taipei Medical University, Taipei 110, Taiwan

**Keywords:** DPPH, human platelets, p38 MAPK, NF-κB, pulmonary thrombosis, rutaecarpine

## Abstract

Platelets play a critical role in arterial thrombosis. Rutaecarpine (RUT) was purified from *Tetradium ruticarpum*, a well-known Chinese medicine. This study examined the relative activity of RUT with NF-κB inhibitors in human platelets. BAY11-7082 (an inhibitor of IκB kinase [IKK]), Ro106-9920 (an inhibitor of proteasomes), and RUT concentration-dependently (1–6 μM) inhibited platelet aggregation and P-selectin expression. RUT was found to have a similar effect to that of BAY11-7082; however, it exhibits more effectiveness than Ro106-9920. RUT suppresses the NF-κB pathway as it inhibits IKK, IκBα, and p65 phosphorylation and reverses IκBα degradation in activated platelets. This study also investigated the role of p38 and NF-κB in cell signaling events and found that SB203580 (an inhibitor of p38) markedly reduced p38, IKK, and p65 phosphorylation and reversed IκBα degradation as well as p65 activation in a confocal microscope, whereas BAY11-7082 had no effects in p38 phosphorylation. The 2,2-diphenyl-1-picrylhydrazyl (DPPH) assay shows that RUT and BAY11-7082 did not exhibit free radical scavenging activity. In the in vivo study, compared with BAY11-7082, RUT more effectively reduced mortality in adenosine diphosphate (ADP)-induced acute pulmonary thromboembolism without affecting the bleeding time. In conclusion, a distinctive pathway of p38-mediated NF-κB activation may involve RUT-mediated antiplatelet activation, and RUT could act as a strong prophylactic or therapeutic drug for cardiovascular diseases.

## 1. Introduction

Traditional Chinese medicine has been used for more than a thousand years in the Asia-Pacific region. Rutaecarpine (RUT, C_18_H_13_N_3_O) is a natural alkaloid isolated from *Tetradium ruticarpum* (also named Wu-Chu-Yu) and is clinically used to treat several conditions, including gastrointestinal disorders, headache, amenorrhea, and postpartum hemorrhage [1]. The anti-hypertensive mechanism of RUT has been proposed by the stimulation of the release of calcitonin gene-related peptide (CGRP), a primary transmitter in capsaicin-sensitive sensory nerves, and CGRP in turn, relaxes the vascular smooth muscle and reduces peripheral resistance [2]. In addition, RUT acts as a natural vasodilator, and its action appears to be dependent on the endothelium and involves nitric oxide (NO) and guanyl cyclase activation [3]. Moreover, RUT can cause the contraction of the atrium, increase the frequency of contraction, and protect the myocardium against ischemia–reperfusion injury [4]. Furthermore, RUT exerts a potent antianoxic effect on the brain anoxia models. Thus, RUT can exert valuable effects on cardiovascular diseases (CVDs) [5].

Platelets, which are anucleated cells, play a crucial role in arterial thrombosis and represent a critical link between inflammation and thrombosis [6]. When blood vessels are damaged, platelets adhere to the disrupted subendothelial matrix, such as collagen. Surface-adherent platelets are activated by various agonists (i.e., collagen, thrombin, arachidonic acid [AA], and ADP) that subsequently release or synthesize some biologically active constituents (e.g., thromboxane A_2_ [TxA_2_], ADP, Ca^2+^, and serotonin) that trigger their aggregations [7]. In addition, the process of platelet activation is accompanied by the production of different inflammatory mediators, namely P-selectin, interleukin (IL)-1β, transforming growth factor-β_1_ (TGF-β_1_), matrix metalloproteinases, tumor necrosis factor (TNF)-α, and IL-6 [8]. The NF-κB signaling pathway contributes a chief role in regulating numerous cellular events, including inflammatory and vascular pathological responses [9]; upon activation, this transcription factor translocates into the nucleus from the cytosol and regulates the respective gene expression. Activated NF-κB enhances the development of unstable coronary plaques, leading to atherosclerosis in humans [10]. Though platelets do not have a nucleus, their activation is still controlled by several functional transcription factors, including signal transducer and activator of transcription 3 (STAT3) and NF-κB [11,12]. During the platelet activation, the activated NF-κB signaling cascades were also observed, such as the inhibitor of κB (IκB) kinase β (IKKβ) phosphorylation, IκBα degradation, and p65 phosphorylation [11,12,13,14]. Conversely, unlike the functioning role of NF-κB signaling in nucleated cells, its role in platelets is still partially understood.

A previous study [15] reported that RUT (40–200 μM) has an inhibitory effect against various agonists that induced platelet aggregation in human platelet-rich plasma (PRP). Recently, Huang et al. [16] discovered that RUT (1–5 μM) strongly suppressed collagen-induced platelet aggregation in washed human platelets by inhibiting phospholipase Cγ2/protein kinase C, mitogen-activated protein kinase (MAPK; p38), and phosphoinositide 3-kinase/Akt/glycogen synthase kinase-3β pathways in cyclic nucleotides/vasodilator-stimulated phosphoprotein-independent mechanisms. Although that study examined the inhibitory mechanism of RUT, it did not investigate the role of NF-κB or its interaction with other signals. Therefore, the current study elucidated the inhibitory mechanisms of RUT in human platelets via regulating NF-κB signaling cascades.

## 2. Results

### 2.1. Relative Activities of RUT with NF-κB Inhibitors in Human Platelet Aggregation and P-Selectin Expression

**Figure 1:**
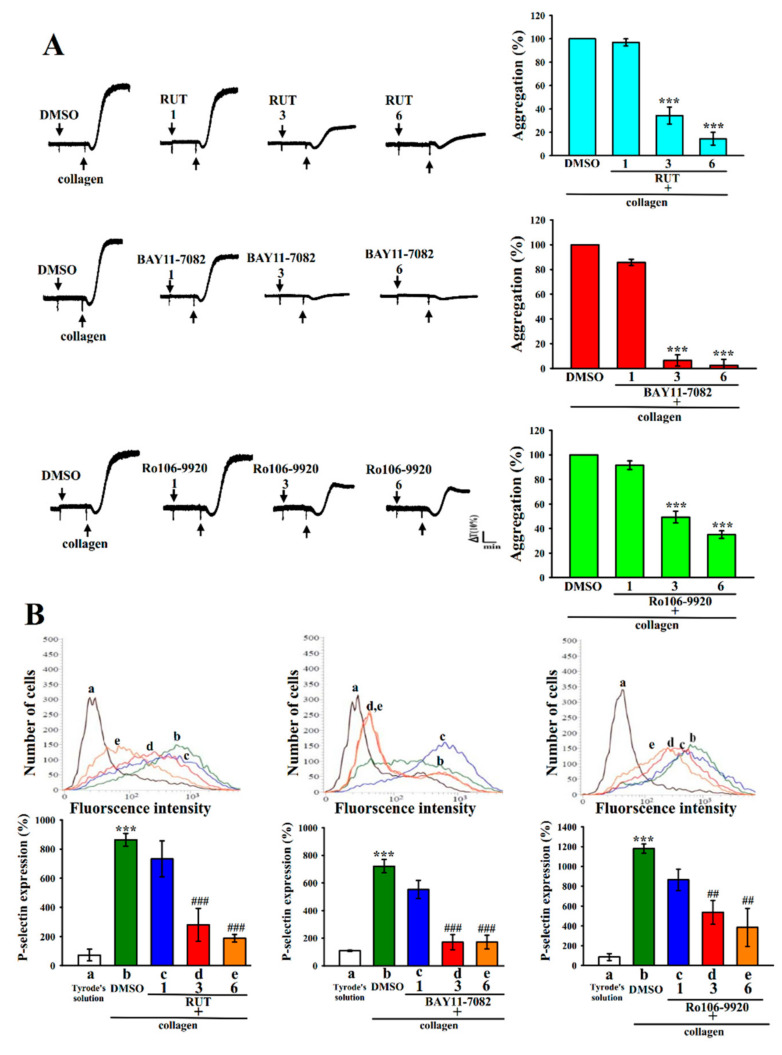
Comparison of the relative activities of rutaecarpine and NF-κB inhibitors in platelet aggregation and P-selectin expression in human platelets stimulated by collagen. Washed human platelets (3.6 × 10^8^ cells/mL) were preincubated with BAY11-7082, Ro106-9920, or rutaecarpine (RUT) at the same concentrations (1, 3, and 6 μM), followed by the addition of collagen (1 μg/mL) to trigger (**A**) platelet aggregation and (**B**) surface P-selectin expression (a, resting controla; b, collagen-activated; c, RUT 1 μM, BAY11-7082 1 μM or Ro106-9920 1 μM; d, RUT 3 μM, BAY11-7082 3 μM or Ro106-9920 3 μM; e, RUT 6 μM, BAY11-7082 6 μM or Ro106-9920 6 μM) as described in the Materials and Methods. The corresponding statistical data are displayed in the right (**A**) or below (**B**) panel of each figure. Data are presented as the mean ± standard error of the mean (*n* = 4). *** *p* < 0.001, compared with the 0.1% dimethyl sulfoxide (DMSO)-treated group (**A**) or resting group (Tyrode’s solution, (**B**)); ^##^ *p* < 0.01 and ^###^ *p* < 0.001, compared with the 0.1% DMSO-treated group (**B**).

BAY11-7082, an IKK inhibitor, has been widely used in anti-cancer, anti-inflammatory, and neuroprotective activities [12,17]. Ro106-9920 is an inhibitor of the ubiquitination of activated IκBα [12,18,19]. Both BAY11-7082 and Ro106-9920 are members of the families of NF-κB inhibitors. In this study, the effect of RUT in collagen-induced platelet aggregation and P-selectin expression in washed human platelets were compared with those of BAY11-7082 and Ro106-9920. BAY11-7082 and Ro106-9920 had a similar effect of RUT on inhibiting collagen (1 μg/mL)-stimulated platelet aggregation in a concentration-dependent manner (1, 3, and 6 μM; Figure 1A). Specifically, on a molar basis, BAY11-7082 showed similar inhibitory activity to RUT, whereas Ro106-9920 proved less activity. The corresponding statistical analysis has given in the right panels of Figure 1A. Moreover, in the normal condition, one of the major biomarkers of platelet activation, P-selectin, is expressed on the inner walls of α-granules; however, in the activated state, the inner walls of the granules expose to the outer parts of cells [14]. As illustrated in Figure 1B, the activity of BAY11-7082 in inhibiting P-selectin expression was similar to that of RUT but higher than that of Ro106-9920. The related statistical data are presented in the below panels of Figure 1B. Therefore, BAY11-7082 was used as an NF-κB inhibitor to determine the functional contribution of NF-κB in RUT-mediated antiplatelet activity in the following experiments.

### 2.2. Regulatory Characteristics of RUT in NF-κB Signals

**Figure 2:**
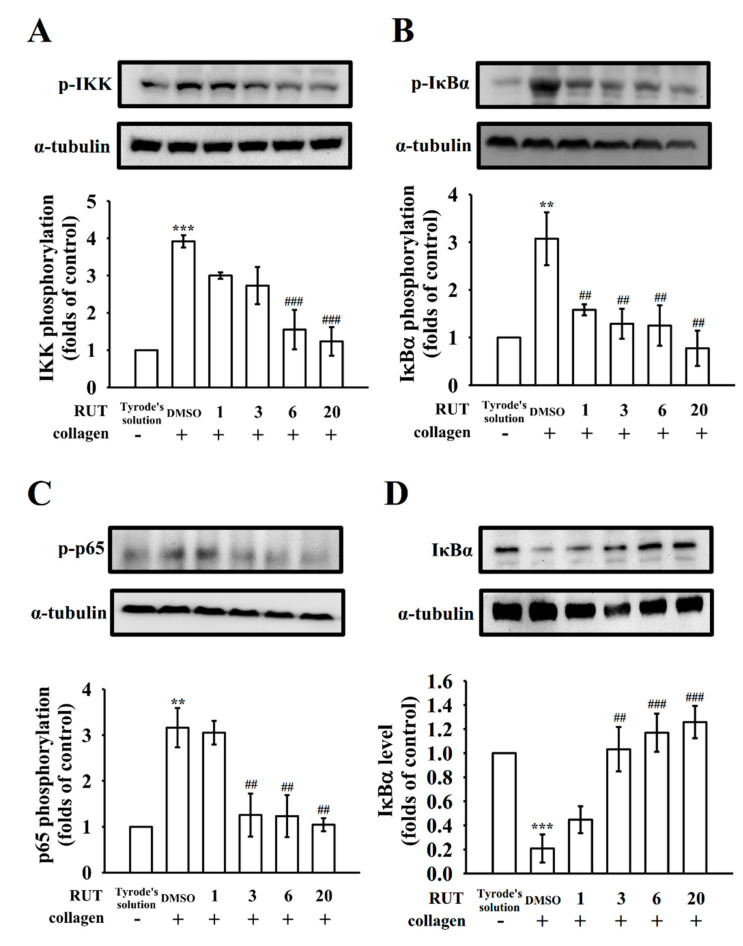
Effects of rutaecarpine on NF-κB activation in platelets. Washed platelets (1.2 × 10^9^ cells/mL) were preincubated with rutaecarpine (RUT; 1–20 μM) or a solvent control (0.1% DMSO), followed by collagen treatment (1 μg/mL) to trigger (**A**) IKK, (**B**) IκBα, and (**C**) p65 phosphorylation as well as (**D**) IκBα degradation for the immunoblotting study. The corresponding statistical data are displayed in the lower panel of each figure. Data are presented as the mean ± standard error of the mean (*n* = 4). ** *p* < 0.01 and *** *p* < 0.001, compared with the resting control (group treated with Tyrode’s solution); ^##^ *p* < 0.01, and ^###^ *p* < 0.001, compared with the 0.1% DMSO-treated group.

NF-κB occurs as an inert cytoplasmic complex by its heterodimer p50 and p65 subunits that are closely bound with IκB [12,20]. Administration of collagen at 1 μg/mL enhanced IKK, IκBα, and p65 phosphorylation as well as IκBα degradation, and RUT (1–20 μM) pretreatment reduced IKK, IκBα, and p65 phosphorylation (Figure 2A–C) and reversed IκBα degradation (Figure 2D) in a concentration-dependent manner. The statistical data are displayed in the below panels of relative Western blotting images. These results indicate that RUT-mediated antiplatelet activity was associated with the inhibition of NF-κB signaling pathways.

### 2.3. Verification of the Signal Connection between p38 and NF-κB

**Figure 3:**
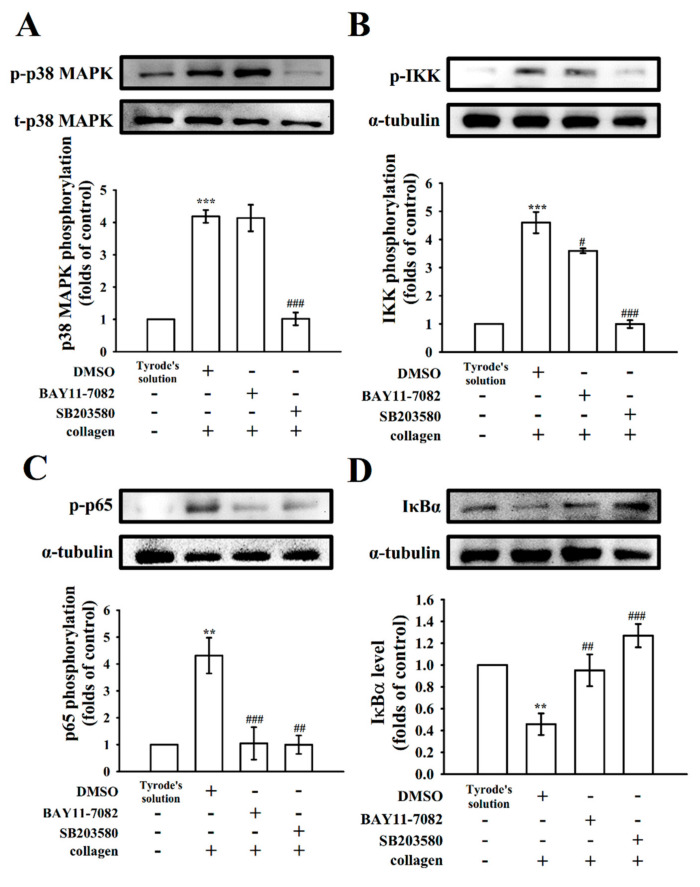
Regulatory role of BAY11-7082 and SB203580 in p38 and NF-κB activation in platelets. Washed platelets (1.2 × 10^9^ cells/mL) were preincubated with a solvent control (0.1% DMSO), BAY11-7082 (6 μM), or SB203580 (20 μM), followed by collagen treatment (1 μg/mL) to trigger (**A**) p38, (**B**) IKK, and (**C**) p65 phosphorylation or (**D**) IκBα degradation for the immunoblotting study. Data are presented as the mean ± standard error of the mean (*n* = 4). ** *p* < 0.01, and *** *p* < 0.001, compared with the resting control (group treated with Tyrode’s solution); ^#^ *p* < 0.05, ^##^ *p* < 0.01, and ^###^ *p* < 0.001, compared with the 0.1% DMSO-treated group.

**Figure 4:**
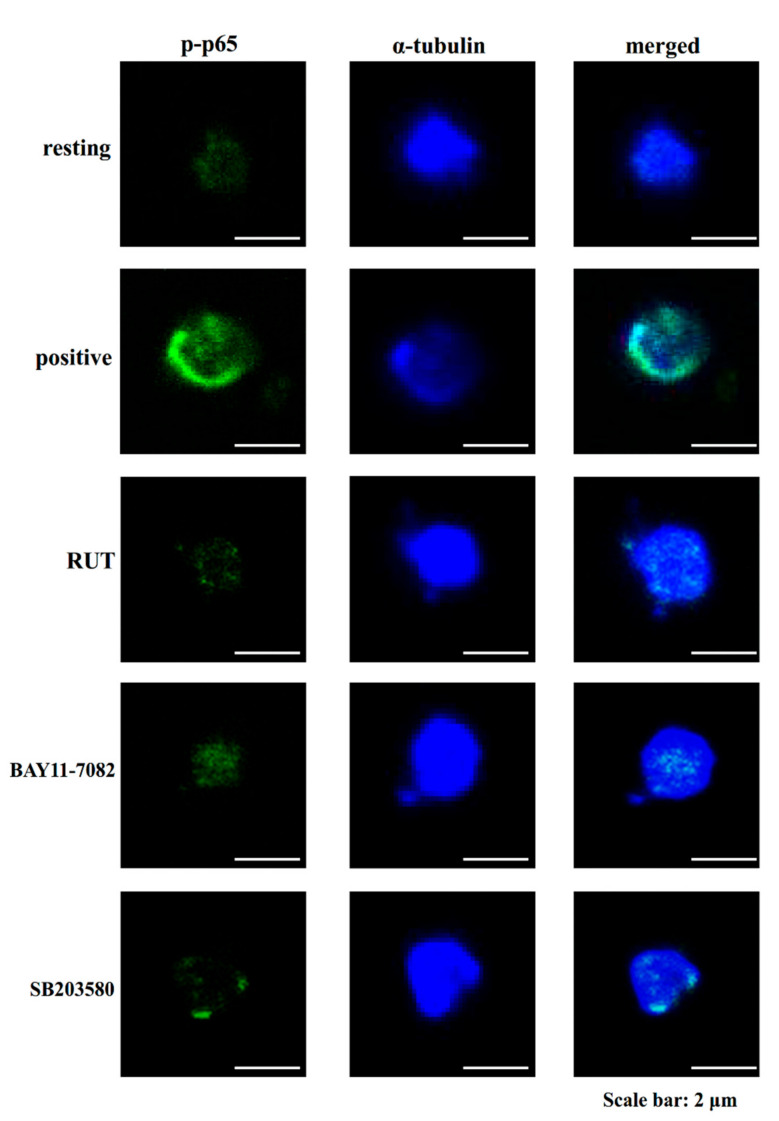
Confocal laser fluorescence of rutaecarpine, BAY11-7082, and SB203580 in the reduction in p65 phosphorylation in human platelets stimulated by collagen. Washed platelets (1.2 × 10^9^ cells/mL) were preincubated with rutaecarpine (RUT; 6 μM), BAY11-7082 (6 μM), or SB203580 (20 μM), followed by the addition of collagen (1 μg/mL) to trigger platelet aggregation. The confocal images of phosphorylated p65 (green fluorescence) and α-tubulin (blue fluorescence) were observed using goat anti-rabbit CF^TM^ 488A Dye and goat anti-mouse CF^TM^ 405M Dye, respectively. The profiles represent four similar studies. Bar: 2 μm.

Mitogen-activated protein kinases (MAPK) are serine and threonine protein kinases that are highly associated with complex cellular events such as platelet activation and cell proliferation, differentiation, and apoptosis. MAPKs including p38, extracellular signal-regulated kinase (ERK)1/2, and Jun *N*-terminal kinase (JNK)1/2 are ubiquitously expressed in platelets [21]. RUT could reduce the collagen-induced p38, ERK1/2, and JNK1/2 phosphorylation, representing that MAPK signaling cascade may be actively involved in the antiplatelet effects of RUT [22]. The functions and mechanisms of p38 in platelet activation are well understood than those of ERK1/2 and JNK1/2. Cytosolic phospholipase A_2_ (PLA_2_) catalyzes the release of AA to produce TxA_2_, a vital substrate of p38 activation that can be induced by platelet agonists [17]. As shown in Figure 3A, SB203580 (20 μM; a p38 MAPK inhibitor), but not BAY11-7082 (6 μM), significantly reduced p38 phosphorylation stimulated by collagen. In addition, both SB203580 and BAY11-7082 nearly abolished IKK and p65 phosphorylation as well as IκBα degradation (Figure 3B–D). Furthermore, the study of confocal microscope indicated that RUT (6 μM), BAY11-7082 (6 μM), and SB203580 (20 μM) markedly reduced p65 activation (green fluorescence) in activated platelets (Figure 4), suggesting that NF-κB seems to be regulated by p38 during platelet activation.

### 2.4. Interaction with Stable Free Radical-Scavenging Action of RUT and BAY11-7082 in a Cell-Free System

**Figure 5:**
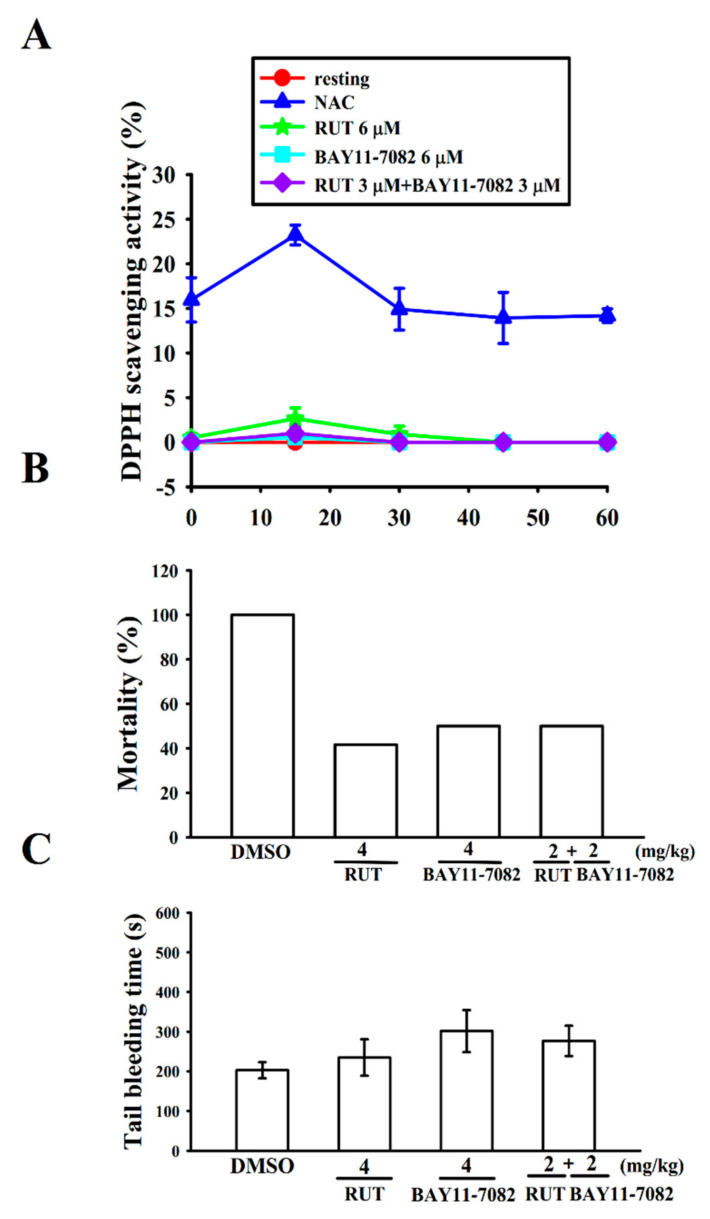
Inhibitory effect of rutaecarpine and BAY11-7082 on free radical formation in the 2,2-diphenyl-1-picrylhydrazyl (DPPH) assay and acute pulmonary thromboembolism as well as tail bleeding time in mice. (**A**) DPPH–methanol (400 μM) was mixed with rutaecarpine (RUT; 6 μM) or BAY11-7082 (6 μM) and then incubated for 30 min. The absorbance at 517 nm was determined using a spectrophotometer. (**B**) Mice were administered an intraperitoneal bolus of the solvent control (0.1% DMSO), RUT (4 mg/kg) or BAY11-7082 (4 mg/kg), or combination of RUT (2 mg/kg) with BAY11-7082 (2 mg/kg), the ADP (700 mg/kg) was then injected into mouse tail vein. The mortality rate of all groups was observed within 10 min after injection. (**C**) The bleeding time was measured through mouse tail transection after 30 min of intraperitoneal administration of the solvent control (0.1% DMSO), RUT (4 mg/kg) or BAY11-7082 (4 mg/kg), or combination of RUT (2 mg/kg) with BAY11-7082 (2 mg/kg). Data are presented as the mean ± standard error of the mean ((**A**), *n* = 4; (**B**,**C**), *n* = 12).

A previous study employed electron spin resonance (ESR) found that RUT could reduce hydroxyl radical formation in activated platelets [16]. However, whether RUT exhibits free radical-scavenging activity under a cell-free system still needs to be verified using the 2,2-diphenyl-1-picrylhydrazyl (DPPH) assay that can be adopted to evaluate the change in absorbance at 517 nm produced by reduced DPPH. As shown in Figure 5A, DPPH decolorization observed in DPPH–methanol (400 μM) increased after treatment with *N*-acetyl l-cysteine (NAC; 20 μM) in a time-dependent manner. Neither RUT (6 μM) or BAY11-7082 (6 μM) alone nor a combination of RUT (3 μM) with BAY11-7082 (3 μM) could significantly reduce the absorbance of DPPH, even at a concentration of RUT up to 100 μM (data not shown), indicating that RUT or BAY11-7082 did not act as a scavenger to interact with the nitrogen-centered stable free radical DPPH.

### 2.5. Relative Potency of RUT and BAY11-7082 in Acute Pulmonary Thrombosis

Huang et al. [16] found a significantly prolonged occlusion time in the RUT administered mesenteric microvessels of mice irradiated by fluorescence. In order to further confirm the clinical application of RUT for treating arterial thrombosis, we examined the effectiveness of RUT in ADP-induced acute pulmonary thrombosis and compared its potency with that of BAY11-7082 (Figure 5B). The results discovered that the ADP (700 mg/kg)-induced mortality rate was reduced by RUT (4 mg/kg) from 100% (12 dead, *n* = 12; 0.1% DMSO control) to 41.6% (5 dead, *n* = 12). Moreover, BAY11-7082 at the same concentration of 4 mg/kg reduced the death rate to 50% (6 dead, *n* = 12), indicating that RUT has significantly high potency than that of BAY11-7082. From these results, it can be designated that RUT is a promising drug candidate for the treatment of thromboembolic diseases. In addition, a combination of RUT and BAY11-7082 at 2 mg/kg, respectively, did not decrease the mortality rate (6 dead, *n* = 12) (Figure 5B).

We compared the bleeding time of the RUT group with that of the BAY11-7082 group because bleeding is a general side effect of antiplatelet drugs observed in clinical trials. The bleeding time was 202 ± 20 s (*n* = 12) in the solvent control (0.1% DMSO) group (Figure 5C). After 30 min of both RUT and BAY11-7082 treatment at 4 mg/kg through intraperitoneal injection, the bleeding times were 234 ± 46 s and 301 ± 53 s (*n* = 12), respectively. Therefore, it indicates that the bleeding time was not significantly affected by those doses. In addition, a combination of RUT and BAY11-7082 at 2 mg/kg, respectively, did not increase the bleeding time (277 ± 38 s; *n* = 12; Figure 5C). Furthermore, after the administration of aspirin (50 mg/kg), the bleeding time was distinctly increased (data not shown).

## 3. Discussion

*Tetradium ruticarpum*, a well-known anti-inflammatory herb, has been used for a long time in the traditional Chinese medical system. Several bioactive alkaloids have been isolated from *Tetradium ruticarpum*, including rhetsinine, wuchuyine, rutaevine, dehydroevodiamine, evodiamine, and RUT [1]. Among these bioactive alkaloids, the most indicative is RUT, which enhances atrial contraction, increases the contraction frequency, protects the heart from ischemia–reperfusion injury, and exerts a hypotensive effect by activating vanilloid receptor subtype 1 [4]. A pharmacokinetic study in rats reported that RUT was speedily absorbed after oral administration, and its Tmax was about 0.5 h [23]. Thus far, only a few studies have determined the plasma concentration of RUT in rats, but no study has examined it in humans [24]. Although evaluation of pharmacokinetics is more challenging in humans compared with animals, it should be explored in future studies. Generally, RUT can be consumed from natural resources; however, it might be inadequate for attaining the plasma concentration, which is required to inhibit in vivo platelet activation; nevertheless, the prolonged consumption of an adequate quantity of RUT can prevent CVDs. Therefore, RUT is an innovative therapeutic drug candidate for t arterial thrombosis-related human diseases.

Studies broadly investigated the significant role of NF-κB in nucleated cells. Free radicals, viral/bacterial infection, or cytokines can stimulate NF-κB activation, which results in inducing inflammation and impairing normal cellular functions [25]. Thus, NF-κB is considered a perfect target against inflammation-related diseases. NF-κB normally occurs in the cytoplasm in an inactive complex; its major isoform is a heterodimer consisting of p50 and p65 subunits. These subunits are tightly bound with IκBα, which is the most represented of the two subunits (IκBα and IκBβ). NF-κB starts activated when IκBα is phosphorylated by the IKK complex. Then, IκBα dissociates from NF-κB subunits, thus resulting in the ubiquitination of IκBα and its rapid degradation by proteasomes associated with NF-κB nuclear translocation [26]. Studies established that several transcription factors expressed in anucleated platelets [27,28] have a nongenomic role. In the present study, RUT could inhibit collagen-stimulated NF-κB activation, indicating that NF-κB signaling may play a key role in RUT-mediated antiplatelet activity. The presence of NF-κB (phosphorylated p65) in the cytoplasm of human platelets was observed through a confocal microscope, indicating its novel cooperation with other signals (i.e., the MAPK pathway) after platelet activation. Moreover, Malaver et al. [29] described that platelet activation could be inhibited by NF-κB inhibitors. Here, NF-κB inhibitor BAY11-7082 showed a stronger effect on inhibiting platelet aggregation and P-selectin expression than Ro106-9920; P-selectin is released from α-granules and considered a marker of platelet activation.

MAPKs are initiated by a successive kinase cascade that mediates several cellular functions from proliferation to apoptosis. MAPKs have three major families, ERKs, JNKs, and p38/stress-activated protein kinases. Activation of ERKs by growth factors regulates the growth and proliferation of cells. JNKs and p38 are activated by inflammatory cytokines and environmental stressors to regulate inflammation, apoptosis, and differentiation [30]. In platelets, p38 is probably the most well-characterized MAPK family that can phosphorylate cPLA_2_ at Ser^505^ [30], producing an increase in TxA_2_ formation. In addition, targeting p38 by SB203580 significantly reduced platelet adhesion, P-selectin expression, and adenosine triphosphate (ATP)/ADP release from granules [30,31], and knockout of p38 attenuated integrin α_IIb_β_3_ activation [32]. In addition, knockout of p38 augmented the survival rate in collagen/epinephrine-induced pulmonary thromboembolism and continued the occlusion time of the carotid artery and tail bleeding time in mice [32,33]. In conclusion, the results highlight the significant role of p38 in controlling hemostasis and thrombosis. This study shows SB203580 reduced NF-κB activation whereas BAY11-7082 had no effects in p38 phosphorylation, signifying that the NF-κB activation may be regulated by p38 in platelet activation, and these signals contribute to the antiplatelet effects of RUT. However, we did not rule out the possible involvement of other unknown mechanisms or signals in this reaction.

DPPH, a stable free radical, has an unpaired electron at one atom of the nitrogen bridge [34]. DPPH scavenging free radical assay is one of the most widely used DPPH antioxidant assays. RUT did not exhibit significant antioxidative activity in the cell-free system but could scavenge hydroxyl radicals in human platelets, as determined using ESR [16]. Hydroxyl radicals are the most reactive free radicals derived during the platelet activation, and it could act as a secondary stimulator in the early phase of platelet activation, thus playing a crucial role in arterial thrombosis [35]. Therefore, the inhibition of in vivo thrombogenesis may involve, at least in part, via the scavenging of free radicals formed in activated platelets. Experimental mouse models are key in understanding the therapeutic implication of test drugs against thrombosis since they are theoretically simple, speedy to operate, and effortlessly reproducible. For instance, in ADP-induced acute pulmonary thromboembolism, platelet aggregation is closely involved in experimental arterial thrombosis. In this study, we found that RUT was potent compared with BAY11-7082 at the same dosage. In addition, heparin (1.5 U/g) exerted no effects on this model, indicating that platelet aggregation rather than fibrin formation is a serious source of thromboembolism in this model.

## 4. Materials and Methods

### 4.1. Materials

RUT (>98%), 6-(phenylsulfinyl)-tetrazolo [1, 5-b] (Ro106-992), 4-[4-(4-fluorophenyl)-2-[4-methylsulfinyl)phenyl]-1H-imidazol-5-yl]-pyridine (SB203580), and (*E*)-3-(4-methylphenylsulfonyl)-2-propenenitrile (BAY11-7082) were obtained from Cayman (Ann Arbor, MI, USA). Collagen (type I), bovine serum albumin (BSA), ethylenediaminetetraacetic acid (EDTA), ADP, paraformaldehyde, DPPH, NAC, and heparin were purchased from Sigma (St. Louis, MO, USA). Anti-IκBα (44D4), anti-phospho-IκBα (Ser^32/36^, 5A5), and phospho-IKKα/β (Ser^176/180^) (16A6) monoclonal antibodies (mAbs), as well as the anti-phospho-NF-κB p65 (Ser536) polyclonal antibody (pAb), were purchased from Cell Signaling (Beverly, MA, USA). The anti-phospho-p38 MAPK (Thr^180^/Tyr^182^) pAb was purchased from Affinity (Cincinnati, OH, USA). The protein assay dye reagent was purchased from Bio-Rad Laboratories Inc. (Hercules, CA, USA). The fluorescein isothiocyanate (FITC)-anti-human CD42P (P-selectin) mAb was obtained from BioLegend (San Diego, CA, USA). CF^TM^488A Dye and CF^TM^405M Dye were obtained from Biotium (Hayward, CA, USA). The anti-α-tubulin mAb was obtained from NeoMarkers (Fremont, CA, USA), and the Hybond-P polyvinylidene difluoridemembrane, enhanced chemiluminescence (ECL) Western blotting detection reagent and analysis system, horseradish peroxidase (HRP)-conjugated donkey anti-rabbit immunoglobulin G (IgG), and sheep anti-mouse IgG were obtained from Amersham (Buckinghamshire, UK). RUT was dissolved in 0.1% DMSO and stored at 4 °C until use.

### 4.2. Platelet Aggregation and Surface P-Selectin Expression

The Institutional Review Board of Taipei Medical University (TMU-JIRB-N201812024) approved this study, and the directives of the Helsinki Declaration were followed. Thirty healthy human blood donors were used to prepare platelet suspensions [36] and mixed with an acid–citrate–dextrose solution (9:1, *v*/*v*). After centrifugation, the platelet-rich plasma (PRP) supplemented with EDTA (2 mM) and heparin (6.4 U/mL) was incubated for 5 min and centrifuged again. The platelet pellets were suspended and centrifuged, finally suspended in Tyrode’s solution containing BSA (3.5 mg/mL). The cells were counted using a Coulter counter (Beckman Coulter, Miami, FL, USA). The final concentration of Ca^2+^ was 1 mM. Washed platelets (3.6 × 10^8^ cells/mL) were untreated or pretreated with solvent control (0.1% DMSO) or RUT, Ro106-992, and BAY11-7082 with the same concentrations of 1, 3, and 6 μM for 3 min before being stimulated with collagen (1 μg/mL) for 6 min. Platelet aggregation was quantified using a Lumi-Aggregometer (Payton, Scarborough, Ontario, Canada), and the turbidimetric method was adopted for measurement [36]. The extent of platelet aggregation was calculated as a percentage of the control (the group treated with Tyrode’s solution) in light transmission units.

For the study of P-selectin expression, washed platelets (3.6 × 10^8^ cells/mL) were preincubated with either solvent control (0.1% DMSO) or RUT, Ro106-992, and BAY11-7082 (1, 3 and 6 μM) and the FITC-conjugated anti-P-selectin mAb (2 µg/mL) for 3 min, followed by stimulation with collagen (1 µg/mL). Afterward, the suspensions were used to assay fluorescein-labeled platelets by using a flow cytometer (FAC Scan system; Becton Dickinson, San Jose, CA, USA). Data were collected from 50,000 platelets in each experimental group, and the platelets were identified based on their characteristic forward and orthogonal light-scattering profiles. Independent experiments (*n* = 4) were performed to ensure the reproducibility of all the experiments.

### 4.3. Immunoblotting

Washed platelets (1.2 × 10^9^ cells/mL) were preincubated with various concentrations of RUT, BAY11-7082, or SB203580, and a solvent control (0.1% DMSO) for 3 min. Subsequently, collagen was added to induce platelet activation. The platelet suspensions were lysed and electrophoretically separated through 12% sodium dodecyl sulphate–polyacrylamide gel electrophoresis. The separated proteins were transferred using the semidry transfer (Bio-Rad, Hercules, CA, USA) and then blocked with TBST (10 mM Tris-base, 0.01% Tween 20, and 100 mM NaCl) containing 5% BSA for 1 h. The membranes were incubated with respective primary antibodies and then with HRP-conjugated anti-mouse or anti-rabbit IgG (diluted 1:5000 in TBST) for 1 h. The optical intensity of protein bands was measured using a video densitometer and Bioprofil BioLight software, version v2000.01 (VilberLourmat, Marne-la-Vallée, France). The relative protein expression was calculated after normalizing it to that of the total protein of interest.

### 4.4. Confocal Laser Fluorescence Microscopy

In brief, resting or collagen-activated platelets were fixed in 4% (*v*/*v*) paraformaldehyde on poly-l-lysine-coated coverslips for 1 h. The coverslips were subjected to 0.1% Triton X-100 and incubated with 5% BSA for 1 h and then stained with either the anti-phospho-NF-κB p65 (Ser^536^) pAb or α-tubulin mAb for 24 h to detect p-p65 and α-tubulin. Furthermore, platelets were incubated with goat anti-rabbit CF^TM^ 488A or anti-mouse CF^TM^ 405M Dye for 1 h and then observed under a confocal microscope (Leica TCS SP5, Mannheim, Germany) by using a 100× oil immersion objective lens.

### 4.5. Measurement of Stable Free Radical Scavenging Activity

The scavenging activity of DPPH radical was tested using a method described in a previous study with minor modifications [34]. Briefly, DPPH–methanol (400 μM) was mixed with either RUT (6 μM) or BAY11-7082 (6 μM) and then incubated for 30 min. The absorbance at 517 nm was determined using a spectrophotometer (UV-160; Shimadzu, Kyoto, Japan). NAC was used as a positive control. DPPH scavenging activity (%) was calculated as (A_2_ − A_1_)/A_2_ × 100%, where A_1_ is the absorbance of RUT or BAY11-7082, and A_2_ is the absorbance of DPPH.

### 4.6. ADP-Induced Pulmonary Thrombosis in Mice

The Affidavit of the Animal Use Protocol from Taipei Medical University (LAC-2019-0365) approved all procedures and protocols. Acute pulmonary thromboembolism was induced as described previously [12,37]. The mice were intraperitoneally injected with either 0.1% DMSO, RUT (4 mg/kg), BAY11-7082 (4 mg/kg), or a combination of RUT (2 mg/kg) with BAY11-7082 (2 mg/kg). After 5 min, ADP (700 mg/kg) was injected into each mouse’s tail vein. The death rate of all the groups was calculated within 10 min after injection.

### 4.7. Tail Bleeding Time

The bleeding time was measured after 30 min of the intraperitoneal injection of RUT (4 mg/kg), BAY11-7082 (4 mg/kg; all 50 μL) or 0.1% DMSO in male ICR mice (20–25 g, aged 5–6 weeks; BioLasco, Taipei, Taiwan). A 3-mm-thick section of the mouse tail was cut and immersed in normal saline directly. The bleeding time was recorded until no sign of further bleeding was observed for at least 10 s.

### 4.8. Statistical Analysis

The data are presented as the mean ± standard error of the mean. Values of *n* refer to the number of experiments that were conducted for different blood donors. Significant differences among the experimental mouse groups were analyzed using one-way analysis of variance (ANOVA) with the Student–Newman–Keuls method as the posthoc test to control for family-wise Type-I error. Variations in the experimental setup were calculated using one-way ANOVA. Significant differences between the groups were determined using the Student–Newman–Keuls method. A *p*-value of <0.05 indicated statistical significance. Statistical analyses were performed using SAS Version 9.2 (SAS Inc., Cary, NC, USA).

## 5. Conclusions

The results of this study demonstrated the presence of a distinctive inhibitory pathway between p38 and NF-κB in RUT-mediated antiplatelet activity in humans. The results indicated that RUT could be used as a prophylactic or clinical therapeutic agent for CVDs. Because inflammatory reactions are associated with increased platelet activation, the inhibition of platelet activation by blocking NF-κB signaling can be considered for treating numerous inflammation-related diseases.

## Data Availability

All data generated or analyzed during this study are included in this published article.

## Sample Availability

Not available.
